# Association between serum concentrations of psychotropic drugs and seizure quality during ECT treatment

**DOI:** 10.1186/s12888-026-07981-7

**Published:** 2026-03-17

**Authors:** Maike Scherf-Clavel, Georg C. Ziegler, Michael von Broen, Sebastian Walther

**Affiliations:** Department of Psychiatry, Psychosomatics and Psychotherapy, Center of Mental Health, University Hospital of Würzburg, 97080 Würzburg, Germany

**Keywords:** Electroconvulsive therapy, Serum concentration, Psychopharmacotherapy, seizure duration, postictal suppression index

## Abstract

**Background:**

Electroconvulsive therapy (ECT) is a highly effective treatment option for treatment-resistant psychiatric disorders. Seizure duration and the postictal suppression index (psi) have been suggested as markers for treatment outcome. As concomitant psychopharmacological medication can affect seizure threshold, understanding its effects on seizure quality could help optimize ECT. The aim of the analyses was to investigate associations between serum concentrations of psychopharmacological drugs and seizure duration and psi.

**Methods:**

A total of 302 ECT sessions in 87 patients were analyzed. Associations between serum concentrations of psychiatric drugs and seizure duration and psi were investigated using multiple linear regression models corrected for age, sex, stimulation side, the interaction number of ECT session:stimulus, as well as seizure-threshold-lowering comedication with clustered standard errors at patient level to account for intra-group correlation.

**Results:**

Four substances demonstrated effects on ECT quality. Norquetiapine serum concentrations were negatively associated with seizure duration, whereas mirtazapine + N-desmethylmirtazapine serum concentrations showed a positive association. Psi was positively associated with sum serum concentrations of amitriptyline and risperidone. Sensitivity analyses point on robust results for quetiapine and amitriptyline.

**Conclusion:**

These findings provide first evidence that serum concentrations of amitriptyline and quetiapine may influence seizure quality, suggesting potential for individualized optimization of ECT parameters. For clozapine, despite its seizure threshold-lowering effect, our data indicate that therapeutic-range concentrations do not increase the risk for excessively prolonged seizures.

**Clinical trial number:**

not applicable.

**Supplementary Information:**

The online version contains supplementary material available at 10.1186/s12888-026-07981-7.

## Background

Electroconvulsive therapy (ECT) is one of the most effective treatment options for treatment-resistant psychiatric disorders [1–3]. For example, in treatment-resistant schizophrenia, ECT efficacy ranges from 40 to 70%, and also in patients with psychotic depression or catatonia, high rates of response to ECT were reported [4, 5]. E.g. in treatment-resistant depression therapeutic response can be reached in two thirds of all patients [6] whereas response rates to antidepressive drugs decrease with each additional trial [7]. ECT treatment is a cost-effective and clinically powerful alternative after one or two failed medication trials to avoid the patient remaining impaired for months [4]. Moreover, also against new treatment options, for example ketamine, ECT remains the gold-standard in the treatment of difficult-to-treat forms of depressive disorders [8]. ECT seems to be equally effective in women and men [9]. Despite its merits, ECT is widely underused [3, 10], although, mild or moderate cognitive side effects usually resolve within days or weeks after ECT treatment [4]. In line, ECT is effective and well tolerated also in geriatric depressed patients regardless of preexisting cognitive impairment [11, 12]. Moreover, in late-life depression, long-term outcome (5-years) of ECT in terms of relapse, cognitive impairment, and survival seems to be in line compared to other treatment options [13]. Also, preliminary insights on long-term safety were provided that did not show an association with an increased risk for dementia [14]. Treatment guidelines for depression or schizophrenia emphasize the use of ECT [15–17]. Clinicians aim to identify subjects who are likely to have the most beneficial effects from ECT.

Previous research on ECT focussed on ictal duration (seizure duration) as therapeutic outcome marker [18]. It has been established, that a longer duration of the seizure was associated with higher ECT efficacy [3, 19]. In a cohort study including 6998 patients, seizure durations from 60 to 69 s led to the highest remission rates, whereas patients with a seizure duration of less than 20 s had the lowest remission rates [19]. Additionally, a longer seizure duration was associated with slower postictal EEG recovery [20]. However, more recently, it was shown that seizure duration alone may not reliably predict therapy outcome, but may have the utility to limit cognitive side effects, if the duration is between 25 and 180 s [18]. Seizure duration is negatively affected by the number of ECT session, as well as by the stimulation frequency [18]. In addition to seizure duration, the postictal suppression index (psi) appears to be associated with a favourable clinical outcome following ECT treatment [3, 18, 21]. In short, the psi is calculated from a quotient between the EEG amplitude shortly after cessation of the epileptiform EEG pattern and mean EEG amplitude during the seizure [22]. Despite its use as a predictor of clinical outcome [18, 21], it has been suggested that only the psi from the first ECT session may predict therapeutic outcome for the ECT series, whereas its predictive power may be limited in the following sessions [22]. Prior studies showed that the psi is, among other factors, affected by the number of the ECT sessions [18]. More recent data reported a prediction model to predict ECT effectiveness (*N* = 1892); the R^2^ of the final model was 19%, including e.g. age, duration of the current episode, antidepressant resistant, pre-ECT cognitive functioning as predictors [23]. However, authors also showed that a large proportion of the variance in depression outcome followed by ECT remained unpredictable [23].

Patients treated with ECT are usually concomitantly treated with psychopharmacological drugs. These drugs, thereby, affect the individual seizure threshold [24]. Dose equivalents of chlorpromazine, and fluoxetine were associated with the seizure threshold [24]. Seizure duration of patients treated with bupropion, sertraline or venlafaxine differed significantly [25]; the longest seizure duration was observed in patients treated with sertraline, followed by venlafaxine and bupropion [25]. Seizure duration between patients treated with the second generation antipsychotics aripiprazole, olanzapine and quetiapine did not differ [25]. In contrast, it was suggested that quetiapine may have seizure reducing properties [26].

There is only a limited number of studies investigating psychopharmacotherapy in association with ECT (e.g. [24, 25]). Notably, no study focused on serum concentrations of these drugs. A deeper understanding of psychopharmacological factors affecting seizure quality may enhance ECT outcomes. We hypothesized that serum concentrations of psychopharmacological drugs may affect seizure duration and psi in a naturalistic clinical setting. Thus, the aim of the present analysis was to investigate associations between serum concentrations of psychopharmacological drugs and seizure duration, as well as the psi.

## Methods

Inpatients of the Department of Psychiatry, Psychosomatics and Psychotherapy, University Hospital of Wuerzburg, Germany, undergoing ECT treatment between November 2023 and May 2025 with available serum concentration determinations of psychiatric drugs were included in the retrospective analysis regardless of diagnosis. ECT sessions were included in the analysis, if therapeutic drug monitoring (TDM) of a psychiatric drug was requested at the day of ECT plus/minus two days. Accordingly, electrolyte concentrations (sodium, potassium) were determined at the day of ECT or within the 4 previous days. Only patients with sodium and potassium within the normal range (sodium: 135–145 mmol/l; potassium: 3.5–5.0 mmol/l) were included to avoid a possible confounding effect due to deviating electrolyte concentrations. Moreover, only patients with bitemporal, or right unilateral electrode placement were included. We chose seizure duration and the psi as seizure quality parameters. The retrospective study was approved by the local ethics committee of the University of Wuerzburg (2025-239-dvhd) and carried out in accordance with the ethical principles of the Helsinki Declaration.

### Therapeutic drug monitoring

Serum concentration determinations were applied according to the AGNP-TDM expert group consensus guideline (trough levels at steady-state) [27]. Daily calibrations and internal quality control samples, integrated in each analytical series, ensure correct analytical results. The laboratory was certified by a quality control program [28].

Dimensional outliers (≥ 3 standard deviation (SD) from mean) of the serum concentration of the relevant active part (parent drug, or active moiety, respectively [27]) for psychiatric effect were set as missing data.

If serum concentrations of a metabolite were available, sum serum concentrations (serum concentration metabolite+serum concentration parent drug), as well as metabolite-to-parent ratios (MPR; serum concentration metabolite/serum concentration parent drug) were calculated [29–31].

### Electroconvulsive therapy

The indication for ECT treatment was made by the professional therapeutic team led by a senior physician with board certification in psychiatry and psychotherapy. Main indications were treatment-resistant major depressive disorder, bipolar depression or schizophrenia, or very severe psychiatric conditions with major harms, e.g. suicidality or catatonia, depression with delusions or depression with severe psychomotor disturbance. Patient preferences were taken into account and the decision for ECT treatment was made only after detailed medical information. Finally, written informed consent for the ECT procedure and the anaesthesia were obtained in all subjects. Before each ECT session, lithium is tapered to 0.4–0.6 mmol/L and paused, and benzodiazepines are reduced, when possible, to ensure adequate excitability. Antipsychotics and other mood stabilizers remain unchanged to allow symptom changes to be attributed to ECT rather than medication adjustments. Anaesthesia was led by a board-certified anaesthesiologist. After establishment of a venous access, placement of ECG monitoring electrodes and preoxygenation anaesthesia was induced by a weight- and age-adjusted dose of i.v. methohexital, and the patient was ventilated by mask ventilation. Muscle relaxation was induced by a weight- and age-adjusted dose of i.v. succinylcholine or rocuronium. Four minutes after anaesthesia induction the seizure was induced by applying an electrical current via two stimulation electrodes placed on the right temple and the d’Elia point [32] for right unilateral stimulation or at both temples for bitemporal stimulation. All ECT treatments were performed with a Thymatron^®^ IV (Somatics, LLC) device with standard presets and the 2X dose programme. The stimulation dose was chosen by the psychiatrist in charge, mostly starting with an age-based dosing regime (initial stimulation dose in % corresponding to the patient’s age in years). Psi was calculated for each ECT session.

### Statistical analyses

Statistical analyses were conducted in R v4.5.0 [33].

Associations between seizure quality criteria (seizure duration, and psi) and serum concentrations (sum concentration or interaction serum concentration metabolite:parent drug), or MPR of the respective drug were calculated using multiple linear regression analyses, corrected for age, sex, stimulation side, the interaction number of ECT session:stimulus, as well as seizure-threshold-lowering comedication, and seizure-threshold-increasing comedication with clustered standard errors at patient level to account for intra-group correlation. Intake of seizure threshold-lowering comedication with high risk (bupropion, clomipramine, clozapine) were included as covariate (factor yes/no) [34, 35]. Moreover, intake of seizure threshold-increasing comedication (lithium, valproic acid, lamotrigine, pregabalin, lorazepam, temazepam, diazepam) were included as covariate (factor yes/no). Exposure to multiple seizure threshold-lowering, or increasing medication was treated as single binary variable. Seizure duration and psi for each ECT session was included, not an average value. Only drugs with a sufficient number of samples (at least *N* = 30 ECT sessions) were analyzed.

A p-value < 0.05 was considered significant. Within each regression model, the p-values for individual predictors were adjusted for multiple testing using the Benjamini–Hochberg False Discovery Rate (FDR) correction. We did not correct for multiple testing across all models (seizure duration and psi: *N* = 25, each); therefore, the results are exploratory with a limited control of type I errors across all medications and both outcomes.

We conducted sensitivity analyses to assess the robustness of the significant association between drug concentrations and outcome parameters in linear regression models; therefore, regression models were restricted to ECT session 1–3.

To control for an association between the serum concentrations, and MPR of the drugs with stimulus and/or number of ECT, a linear regression model including serum concentrations, and MPR as outcome variable and the interaction stimulus:number of ECT session as confounding parameter with clustered standard errors at patient level was calculated. If applicable, ROC analysis to determine a threshold in serum concentrations to predict a seizure duration less than 20 s [19] was conducted, using the R packages “ROCit” and “pROC” [36, 37].

## Results

### Sample

Eighty-seven patients were included in the retrospective sample. In these patients, data of 302 ECT sessions with simultaneous serum concentration determinations were available. From these 87 patients 49 were male (56.3%), and 38 female (43.7%); at the date of their first ECT session patients were 51.8 ± 17.6 years old (mean±standard deviation (SD); range 19–81 years).

ECT relevant diagnoses in the 87 patients were affective disorders (*N* = 69): 52 with major depressive disorder (including 9 with psychotic features) and 18 with bipolar disorder (8 in a depressive episode, 6 depressive with psychotic features, 1 manic (rapid cycling), 2 manic with psychotic features, and 1 in a mixed state). The remaining 18 patients had schizophrenia-spectrum disorders, including 9 with schizoaffective disorder (7 depressive, 2 mixed states), 6 with catatonic schizophrenia, 2 with hebephrenic, and 1 with paranoid schizophrenia, according to ICD-10 criteria. For a full summary of all comorbidities see supplemental Table 1.

Across all ECT sessions, patients received between 1 and 19 drugs (mean ± SD 6.8 ± 3.3); the most prevalent drugs were quetiapine (*N* = 173), amitriptyline (*n* = 155), and risperidone (*n* = 119). A full summary of all drugs is given in supplemental Table 2.

The mean number of ECT sessions per patient included in the sample was 3.85 (SD 2.82; range 1–14); bitemporal stimulation was applied in 85 sessions, whereas right unilateral stimulation was used in 217 sessions. Across all ECT sessions, mean stimulation intensity was 128.0%±68.5% (mean ± SD; range 10%-200%).

Mean seizure duration across all included ECT sessions was 37.4 ± 22.1 s (mean ± SD; range 0–128 s). Extremely short seizure durations (0–10 s) went along with a high stimulus (200%) in 8 out of 10 ECT sessions; in two cases stimulus was 75% and 100% respectively. Restimulation was not done in patients included in our sample. In 222 ECT sessions, the psi was available with a mean ± SD of 70.0 ± 22.6% (range 10–99%). A summary of patients and ECT sessions included in each model (seizure duration and psi) is given in supplemental Table 3.

Seizure quality criteria with according serum concentrations determinations with a sufficient number of samples were available for amitriptyline (ami; *N* = 130), quetiapine (quet; *N* = 106), risperidone (risp; *N* = 77), venlafaxine (ven; *N* = 68), mirtazapine (mirt; *N* = 66), clozapine (cloz; *N* = 42), olanzapine (olanz; *N* = 41), aripiprazole (ari; *N* = 40), doxepin (dox; *N* = 30), and cariprazine (cari; *N* = 30) (supplemental Table 4). Due to power reasons, only in these groups statistical analyses targeting seizure criteria were calculated.

Mean (± SD; range) seizure duration, as well as psi and serum concentrations are summarized in Table 1.

### Amitriptyline

After excluding outliers, serum concentrations of ami were available in 126 ECT-sessions across 33 patients.

Neither sum serum concentration of ami, nor ami concentration, nor nortriptyline concentration, nor MPR was associated with stimulus and/or number of ECT sessions.

Seizure duration was not associated with sum serum concentration, serum concentration of ami, serum concentration of nor, and the MPR. However, in the model including MPR, seizure duration was associated with the stimulus (for details see Table 2); an increased stimulus was associated with a shorter seizure duration.

**Fig. 1:**
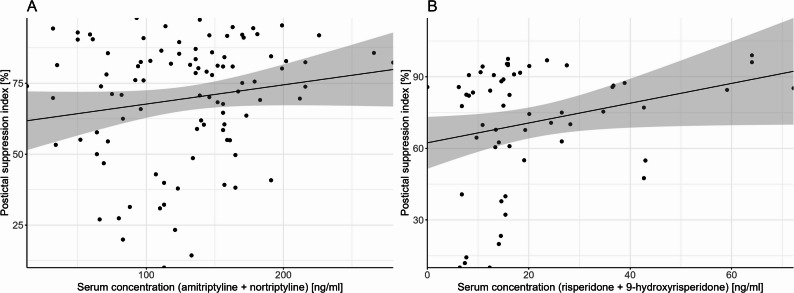
Association between the postictal suppression index and sum serum concentration amitriptyline (**A**), and the interaction serum concentration of olanzapine:serum concentration N-desmethylolanzapine (**B**). The psi increased with increasing sum serum concentrations of amitriptyline. In contrast, the psi decreased with higher serum concentrations of olanzapine, particularly in individuals with lower serum concentrations of N-desmethylolanzapine

The psi was positively associated with the sum serum concentration of amitriptyline (ami + nor) (*p* = 0.003; ß=0.11; 95% confidence interval (CI) [0.05; 0.18]; Fig. 1), but neither with serum concentration of ami or nor, nor with the MPR. However, stimulation side was associated with psi in all models; moreover, the number of ECT sessions were associated with the psi in the model including sum serum concentration, and age was associated with psi in the model including sum serum concentration and MPR (for details see Table 3). Therefore, with increasing age and increasing number of ECT session the psi decreased; moreover, patients stimulated bitemporally showed an increased psi compared to patients stimulated right unilateral.

Sensitivity analysis concerning sum serum concentration and psi yielded a result consistent with the main model (*p* = 0.04; ß=0.09; 95% confidence interval (CI) [0.003; 0.17]).

**Table 1:** Mean (± standard deviation (sd), range) seizure duration, postictal suppression index (psi) and serum concentrations in the respective drug groups (cParent, cMetabolite, cSum). MPR, Metabolite-to-parent ratio

	Mean (sd; range)					
	Seizure duration [sec]	psi [%]	cParent [ng/ml]	cMetabolite [ng/ml]	cSum [ng/ml]	MPR
Amitriptyline	36.5 (22.6; 0-128)	69.6 (21.3; 10–98)	64.0 (28.7; 5-186)	63.0 (29.0; 8-144)	127.0 (50.7; 13–280)	1.05 (0.43; 0.33–2.63)
Quetiapine	39.0 (25.7; 9-128)	71.2 (25.2; 10-97.5)	97.3 (65.1; 10–339)	86.9 (66.3; 10–293)	184.2 (111.6; 23–529)	1.1 (0.94; 0.14–5.08)
Risperidone	43.4 (27.6; 8-128)	70.3 (25.1; 10–99)	9.97 (11.36; 1.8–49)	11.78 (8.66; 2.3–38.7)	21.75 (15.88; 6.2–72.2)	2.2 (2.1; 0.25–10.97)
Venlafaxine	37.3 (21.8; 7-114)	73.5 (21.0; 10–99)	137.9 (93.4; 9-362)	190.6 (91.2; 35–433)	330.7 (146.8; 51–751)	2.12 (2.12; 0.37–11.56)
Mirtazapine	35.8 (22.2; 8-115)	69.9 (24.8; 10-97.5)	38.4 (23.6; 6 -108)	19.6 (11.7; 4–63)	58.0 (33.2; 11–161)	0.55 (0.19; 0.24–0.96)
Clozapine	39.3 (26.5; 8-100)	73.1 (24.3; 24.9–96.9)	373.7 (160.2; 112–830)	207.6 (93.4; 59–404)	581.3 (242.8; 171–1097)	0.57 (0.11; 0.28–0.75)
Olanzapine	42.2 (21.5; 11–92)	70.3 (17.6; 26-95.3)	49.0 (26.6; 6-125)	8.7 (5.2; 0–23)	61.7 (28.2; 10–148)	0.19 (0.13; 0-0.67)
Aripiprazole	40.7 (22.0; 0-100)	71.0 (20.3; 23.3–96.3)	224 (133; 51–463)			
Doxepin	30.2 (15.7; 7–78)	68.1 (20.7; 26-90.7)	42.6 (18.8; 11–91)	63.3 (36.8; 14–173)	105.9 (49.6; 31–231)	1.53 (0.64; 0.73–2.98)

**Table 2:** p-values (FDR corrected), ß, and 95% confidence intervals of the significant covariates in the linear regression models with seizure duration as outcome variable with clustered standard errors at patient level

Drug	Covariate	Regression model 1 (sum serum concentration)			Regression model 2 (parent drug and metabolite concentration)			Regression model 3 (MPR)		
		*p*-value	ß	95% CI	*p*-value	ß	95% CI	*p*-value	ß	95% CI
amitriptyline	stimulus [%]							0.05	-0.182	-0.32, -0.05
quetiapine	sex	0.02	-18.24	-30.75, -5.73	0.007	-19.50	-31.5, -7.52	0.05	-15.14	-27.07, -3.21
	stimulus [%]	0.001	-0.24	-0.34, -0.12	0.002	-0.24	-0.36, -0.11	0.01	-0.23	-0.38, -0.09
	intake of seizure threshold increasing comedication	0.03	11.68	2.43, 20.93						
	sum serum concentration	0.03	-0.07	-0.13, -0.02						
	norquetiapine serum concentration				0.02	-0.20	-0.34, -0.06			
mirtazapine	intake of seizure threshold-lowering comedication	0.02	14.49	4.53, 24.45						
	sum serum concentration	0.003	0.23	0.11, 0.34						
olanzapine	interaction number of ECT session: stimulus [%]	0.005	-0.04	-0.05, -0.02	0.02	-0.04	-0.06, -0.02	0.01	-0.03	-0.05, -0.01
	intake of seizure threshold increasing comedication	0.03	-18.09	-30.87, -5.30						
aripiprazole*	interaction number of ECT session: stimulus [%]	0.01	-0.04	-0.06, -0.02						
	stimulation side	0.01	41.02	14.83, 67.22						
	intake of seizure threshold-lowering comedication	0.04	-31.96	-58.00, -5.93						

*for aripiprazole only one model including serum concentration of aripiprazole was calculated as no metabolite was determined. MPR, Metabolite-to-parent ratio

**Table 3:** p-values (FDR corrected), ß, and 95% confidence intervals of the significant covariates in the linear regression models with the postictal suppression index as outcome variable with clustered standard errors at patient level

Drug	Covariate	Regression model 1 (sum serum concentration)			Regression model 2 (parent drug and metabolite concentration)			Regression model 3 (MPR)		
		*p*-value	ß	95% CI	*p*-value	ß	95% CI	*p*-value	ß	95% CI
amitriptyline	age	0.04	-0.351	-0.64, -0.07				0.007	-0.44	-0.72, -0.17
	number of ECT session	0.05	-6.89	-12.77, -1.00						
	stimulation side	< 0.001	-20.74	-27.80, -13.68	< 0.001	-20.68	-27.88, -13.48	< 0.001	-19.40	-27.28, -11.53
	sum serum concentration	0.003	0.11	0.05, 0.18						
quetiapine	age	< 0.001	-0.82	-1.21, -0.42	< 0.001	-0.80	-1.21, -0.40	0.006	-0.69	-1.06, -0.31
	stimulation side	0.02	-11.52	-20.59, -2.46	0.04	-11.55	-21.01, -2.08	0.03	-11.96	-21.58, -2.34
	interaction number of ECT session: stimulus [%]	0.003	0.05	0.02, 0.08	0.004	0.05	0.02, 0.08	0.03	0.04	0.008, 0.07
	intake of seizure threshold-lowering comedication	0.01	24.41	7.49, 40.92	0.01	24.8	8.10, 41.59	0.02	24.83	7.09, 42.57
risperidone	age	0.05	-0.65	-1.18, -0.12	0.03	-0.73	-1.25, -0.20			
	stimulation side	0.04	-18.89	-32.50, -5.28	0.005	-26.70	-39.10, -14.31	0.007	-24.19	-38.51, -9.88
	sum serum concentration	0.05	0.44	0.08, 0.81						
mirtazapine	age	0.03	-1.57	-2.60, -0.55						
	stimulus [%]	< 0.001	-20.60	-26.61, -14.58				< 0.001	-22.81	-28.48, -17.15
	stimulation side	0.04	0.05	0.01, 0.09						
clozapine	age	0.04	-0.351	-0.64, -0.07				0.007	-0.44	-0.72, -0.17
	intake of seizure threshold-lowering comedication	0.05	-6.89	-12.77, -1.00						
olanzapine	interaction number of ECT session: stimulus [%]	< 0.001	-20.74	-27.80, -13.68	< 0.001	-20.68	-27.88, -13.48	< 0.001	-19.40	-27.28, -11.53

*for aripiprazole only one model including serum concentration of aripiprazole was calculated as no metabolite was determined. MPR, Metabolite-to-parent ratio

### Quetiapine

After excluding outliers, serum concentrations of quet were available in 103 ECT-sessions across 38 patients.

Number of ECT sessions was positively associated with sum serum concentration, as well as with quet serum concentration, but not with with metabolite concentration or MPR. No factor was associated with stimulus.

**Fig. 2:**
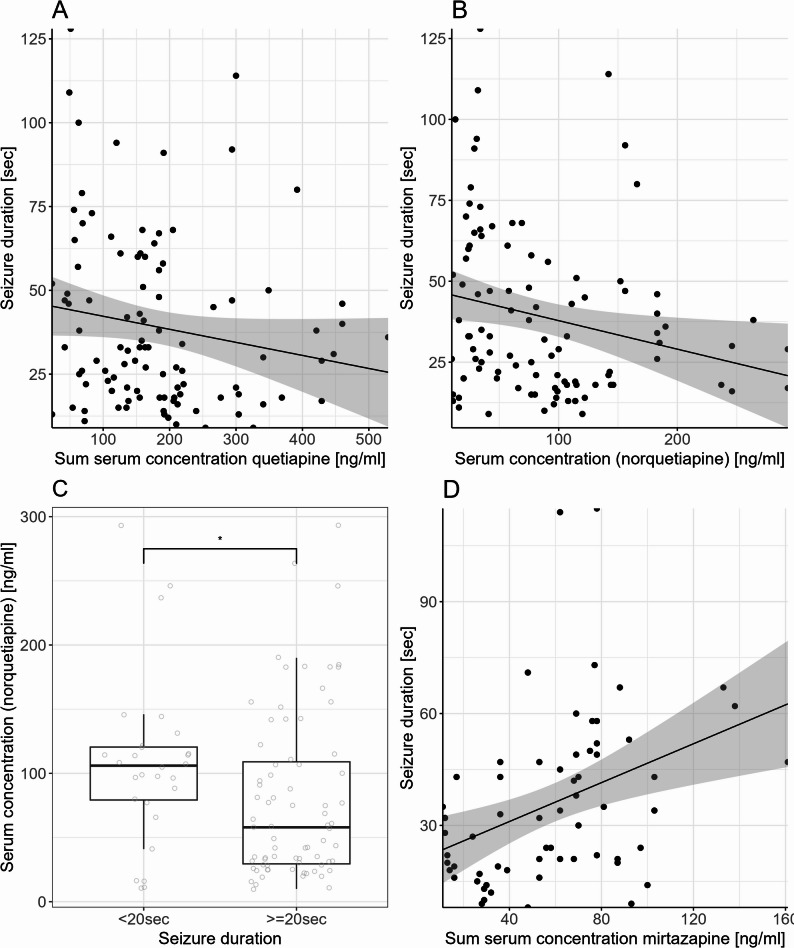
Seizure duration decreased with increasing sum serum concentration of quetiapine (**A**), as well as with increasing concentrations of norquetiapine (**B**); serum concentration of norquetiapine differed between patients with seizure duration < 20 s vs. ≥20 s (Mann-Whitney U test, *p* = 0.03) (**C**). Seizure duration increased with increasing sum serum concentrations of mirtazapine (**D**)

Seizure duration was associated with the sum serum concentration of quetiapine (*p* = 0.03; ß=-0.07; 95% CI [-0.13, -0.02]; Fig. 2), as well as with the serum concentration of norquetiapine (*p* = 0.02; ß=-0.20; 95% CI [-0.34, -0.06]; Fig. 2). Seizure duration, therefore, decreased with increasing serum concentrations. Moreover, female sex, as well as an increased stimulus was associated with a shorter seizure duration in all models; for details see Table 2.

Sensitivity analysis concerning sum serum concentration indicated that the association between sum serum concentration and seizure duration was not robust; the result just missed significance (*p* = 0.06) when including only ECT number 1–3. In contrast, sensitivity analysis regarding metabolite concentration yielded a result consistent with the main model (*p* = 0.04; ß=-0.25; 95% confidence interval (CI) [-0.48; -0.02]).

Assuming a threshold of 20 s for treatment response using seizure duration as predictor [19], serum concentration of norquetiapine was significantly higher (Mann-Whitney U test; *p* = 0.03) in patients with a seizure duration less than 20 s (mean ± sd; 107.6 ± 66.2 ng/ml) compared to patients with a seizure duration ≥ 20 sect. (79.2 ± 19.7 ng/ml; Fig. 2).

The threshold in the serum concentration of norquetiapine to predict seizure duration < 20 s was computed as 76 ng/mL (*p* = 0.03; Youden index: 0.386, specificity 78.6%, sensitivity 60.0%). The area under the ROC curve was 0.641 (95% CI 0.516–0.765) (supplemental Fig. 1).

The psi was not associated with the sum serum concentration of quetiapine, nor with serum concentration of quet or norquetiapine, nor with MPR.

However, age, the interaction number of ECT session:stimulus, stimulation side, as well as intake of seizure threshold-lowering comedication were associated with psi in all models (for details see Table 3). Therefore, with increasing age, and intake of seizure threshold-lowering comedication the psi decreased; moreover, patients stimulated bitemporally showed an increased psi compared to patients stimulated right unilateral. Moreover, an increased number of ECT sessions was associated with increased psi with higher stimulus, in contrast, a lower number of ECT sessions was associated with decreasing psi with higher stimulus.

### Risperidone

After excluding outliers, serum concentrations of risp were available in 65 ECT sessions across 26 patients.

Number of ECT sessions was positively associated with sum serum concentration of risp, and with 9-hydroxyrisperidone concentration, but not with risp concentration and MPR. No factor was associated with stimulus.No covariate was associated with seizure duration in any of the models.

The psi was positively associated with the sum serum concentration of risp (*p* = 0.05; ß=0.44; 95% CI [0.08, 0.81]; Fig. 1), but not with serum concentration of risp or 9-hydroxyrisperidone, nor with the MPR. Moreover, stimulation side was associated with psi in all models (for details see Table 3) with patients stimulated bitemporal showing an increased psi compared to patients stimulated right unilateral. Age was negatively associated with psi in the model including sum serum concentration and serum concentration of risp or 9-hydroxyrisperidone (for details see Table 3).

Sensitivity analysis concerning sum serum concentration and psi indicated that the association was not robust; the result missed significance (*p* = 0.73) when including only ECT number 1–3.

### Venlafaxine

After excluding outliers, the dataset with available venlafaxine serum concentrations included 68 ECT sessions across 22 patients.

Neither sum serum concentration of ven, nor ven concentration, nor O-desmethylvenlafaxine concentration, nor MPR was associated with stimulus and/or number of ECT sessions.

No covariate was associated with seizure duration or the psi in any of the models (sum concentration, ven concentration:O-desmethylvenlafaxin concentration, MPR).

### Mirtazapine

After excluding outliers, serum concentrations of mirt were available in 64 ECT sessions across 22 patients.

Neither sum serum concentration of mirt, nor mirt concentration, nor metabolite concentration, nor MPR was associated with stimulus and/or number of ECT sessions.

Seizure duration increased with increasing sum serum concentration (*p* = 0.003; ß=0.23; 95% CI [0.11; 0.34]) (Fig. 2). Seizure duration was not associated with mirt serum concentration, or the metabolite concentration, nor the MPR. However, sensitivity analysis indicated that the association was not robust; the result missed significance (*p* = 0.71) when including only ECT number 1–3. In the model including sum serum concentration the intake of seizure threshold-lowering comedication was significantly associated with longer seizure duration (for details see Table 2). No covariate was associated with psi in any of the models.

### Clozapine

After excluding outliers, serum concentrations of cloz were available in 41 ECT sessions across 7 patients.

Sum serum concentration of clozapine, as well as clozapine concentration and metabolite concentration, but nor MPR was associated with the interaction number of EKT:stimulus.

No covariate was associated with seizure duration in any of the models (sum concentration, cloz concentration: N-desmethylclozapin concentration, MPR).

The psi was not associated with the sum serum concentration of cloz, nor with serum concentration of cloz or norclozapine, nor with MPR. However, age was associated with psi in the model including sum serum concentration, and intake of seizure threshold-lowering comedication was associated with psi in the model including MPR (for details see Table 3). Therefore, with increasing age and no intake of additional seizure threshold-lowering comedication the psi decreased.

### Olanzapine

After excluding outliers, the dataset with available olanzapine serum concentrations included 41 ECT sessions across 15 patients.

Neither sum serum concentration of olanz, nor olanz concentration, nor metabolite concentration, nor MPR was associated with stimulus and/or number of ECT sessions.

Seizure duration was not associated with olanz serum concentration, or the metabolite concentration, nor the MPR. However, the interaction number of ECT session:stimulus was associated with seizure duration in all models (for details see Table 2). Therefore, an increased number of ECT sessions was associated with decreased seizure duration with higher stimulus, in contrast, a lower number of ECT sessions was associated with increasing seizure duration with higher stimulus.

The psi was not associated with the sum concentration, with the serum concentration of olanz, and N-desmethylolanzapine and not with MPR. In addition, the interaction number of ECT session:stimulus was associated with psi in all models (for details see Table 3). Therefore, an increased number of ECT sessions was associated with increased psi with higher stimulus, in contrast, a lower number of ECT sessions was associated with decreasing psi with higher stimulus.

### Aripiprazole

After excluding outliers, serum concentrations of aripiprazole were available in 40 ECT sessions across 18 patients.

Aripiprazole serum concentration was not associated with stimulus and/or number of ECT sessions.

As no metabolite was determined, regression model only was calculated using the serum concentration of aripip.

Serum concentration of aripip was not associated with seizure duration or psi. However, stimulation side with associated with seizure duration with longer seizure duration in patients stimulated right unilateral, compared to bitemporal stimulation, and the interaction number of ECT session:stimulus was associated with seizure duration. Therefore, the stimulus was associated with increased seizure duration, particularly in the first ECT sessions (for details see Table 2). Moreover, intake of seizure threshold-lowering comedication was associated with seizure duration. Patients taking seizure threshold-lowering comedication showed longer seizure duration compared to patients not taking seizure threshold-lowering comedication (for details see Table 2).

### Doxepin

After excluding outliers, serum concentrations of doxepin were available in 30 ECT sessions across 10 patients.

Neither sum serum concentration of doxepin, nor doxepin concentration, nor metabolite concentration, nor MPR was associated with stimulus and/or number of ECT sessions.

As only one patient with seizure threshold-lowering comedication was included in the doxepin sample, comedication was not included in the regression analyses. No covariate was associated with seizure duration or psi in any of the models (sum concentration, doxepin concentration:N-desmethyldoxepin concentration, MPR).

## Discussion

This retrospective study tested whether serum concentrations of psychoactive drugs would influence the quality of ECT in patients with severe mental illness. In our sample, sum serum concentration of quetiapine, serum concentrations of norquetiapine and sum serum concentrations of mirtazapine were associated with seizure duration during ECT treatment. Seizure duration decreased with increasing quetiapine sum serum concentrations, and norquetiapine serum concentrations and increased with increasing mirtazapine sum serum concentrations. Regarding the postictal suppression index (psi), the sum serum concentrations of amitriptyline, and the sum serum concentration of risperidone were associated with the psi. The psi increased with increasing sum serum concentrations of ami and risp.

In a study by Mohamad et al. (2024) seizure duration during ECT in patients treated with quetiapine did not differ from those treated with aripiprazole or olanzapine [25]. However, quetiapine dosage might vary substantially and neither data on quetiapine dose or serum concentrations in relation to seizure parameters during ECT were available. Thus, for the first time, we report a negative association between sum serum concentration of quetiapine, and serum norquetiapine concentration and seizure duration. In contrast, neither quetiapine serum concentration, nor the MPR was associated with seizure duration. Thus, the norquetiapine serum concentration is the driving factor for the association with seizure duration. However, sum serum concentration was associated with number of ECT sessions; therefore, estimates for this variable should be interpreted with caution. Moreover, sensitivity analyses on sum serum concentration indicated that the result may not be robust. In contrast, sensitivity analysis on the metabolite’s concentration yielded a consistent result. As seizure duration may be essential for treatment response to ECT [3, 19], higher serum concentrations of norquetiapine may be counteracting treatment effectivity. However, standardized clinical outcome measures were not available for our retrospective naturalistic study restricting the significance of our results to technical seizure parameters. In line with our results, Gazdag et al. (2004) reported that quetiapine may have seizure reducing properties [26]. Quetiapine is mainly metabolized by the cytochrome P-450 enzyme (CYP) 3A4 to norquetiapine [38]. Notably, norquetiapine, but not quetiapine inhibits the norepinephrine transporter and muscarinic receptors [38]. Hence, norepinephrine transporter inhibition may be responsible for the possible seizure lowering effect: increased norepinephrine concentrations may increase the seizure threshold [39] by activation of α1-, α2-, and β2-adrenoreceptors [40].

Using ROC analysis, we identified a norquetiapine serum concentration threshold of 76 ng/ml, above which seizure durations of < 20 s were likely to occur. However, both specificity and sensitivity were insufficient for clinical use of this threshold, as it does not reliably distinguish between seizure duration ≥ 20 s and < 20 s. Moreover, the AUC of 0.6405 (indicating sufficient diagnostic accuracy [41]), and a Youden index of 0.386 further argue against its clinical use. Nevertheless, this concentration can be seen as preliminary evidence that attention should be paid to a sufficiently long seizure duration in patients treated with quetiapine.

Pharmacotherapy with mirtazapine is considered safe in combination with ECT [42] and it was suggested as an optional treatment for ECT-induced nausea and headache [43]. For its antidepressant use, the serum concentration of mirtazapine, but not the sum serum concentration is relevant [27]. In contrast, seizure duration was associated with the sum serum concentration of mirtazapine (mirt + N-desmethylmirtazapine), with longer durations observed at higher concentrations. In line with this finding, a previous study reported longer ECT seizure durations in patients treated with mirtazapine as compared to venlafaxine [44]. However, serum concentrations were not evaluated in this study [44]. Our study is the first to report a possible association between mirtazapine sum serum concentration and seizure duration during ECT. However, sensitivity analysis showed that the association was not robust. In general, mirtazapine is not known for seizure-threshold lowering properties and is consequently recommended in patients with epilepsy [45]. Thus, this finding and its clinical relevance should be confirmed in a larger patient sample, before definitive clinical conclusions can be drawn. Previously, lower psi values were observed in patients treated with mirtazapine compared to those treated with venlafaxine or tricyclic antidepressants during ECT [44]. Based on the assumption that mirt enhances the clinical effectiveness of ECT it was suggested that the psi may not be the most reliable parameter for assessing the clinical effectiveness of a single ECT session [44]. This aligns with our results, as mirt concentrations were not associated with psi, but with seizure duration. However, we did not evaluate clinical outcomes in our sample.

In addition to the results on seizure duration, we also investigated associations with the psi, as this index more recently was proposed to potentially predict improvement in clinical outcome following the first ECT session [3, 18, 21, 22]. In our sample, the psi increased with higher sum serum concentrations of amitriptyline, and risperidone. To date, no study has specifically examined the association between amitriptyline, or risperidone and the psi. Thus, these are the first data providing evidence of serum concentration-dependent alterations in seizure quality. Nevertheless, in the risperidone model, sum serum concentration was associated with the number of ECT sessions. Considering multicollinearity and a p-value of 0.05, this association may not be considered true. Also, sensitivity analyses showed that the association was not robust.

In contrast to the known seizure threshold lowering effects of clozapine making seizures more likely during treatment with clozapine [34, 35, 46], we did not find serum concentration dependent associations with either seizure duration, or with psi. In our sample, 56.1% of the measured serum concentrations were within the therapeutic reference range (TRR) (350–600 ng/ml [27], 41.5% were below the TRR and only 1 TDM request (2.4%) was above the TRR). In consequence, it is likely that when patients were treated with serum concentrations below and within the TRR no additional risk for prolonged seizure duration is present.

For clinical use, we suggest monitoring serum concentrations not only to improve the drug´s effectivity and safety [27], but also to potentially improve and personalize ECT treatment.

### Strengths and limitations

This is the first study to examine if and how serum concentrations of psychopharmacological drugs affect seizure duration and the psi. Our retrospective analysis provides real-life data from a naturalistic setting, making the results relevant for clinical practice. However, there are limitations. The number of patients included in each analysis was limited but still provided sufficient data on drug serum levels. Furthermore, standardized clinical outcome data were not available in our retrospective cohort and should be included in clinical routine to help interpret the clinical relevance of TDM findings in connection with ECT. Also, individual treatment courses, for example change of medication or doses were not considered. While this may limit our interpretation, these limitations do not affect the results on the association between serum concentrations and seizure duration and postictal suppression index. We were unable to properly address all issues related to polypharmacy. Due to the complexity of clinical routine data, data on patient-specific drug combinations (1–19 comedications), or doses of comedications were not considered. However, we included high risk seizure threshold-lowering comedication, as well as seizure threshold increasing comedication as confounders in the analyses. Exposure to more than one high risk seizure-lowering comedication (true for 8 patients) was not considered. Moreover, exposure to multiple seizure-increasing comedication (true for 22 patients) was not considered. Moreover, for some of the commonly used psychopharmacological agents our database had too few entries to calculate the association with ECT parameters. We included sensitivity analyses to prove robustness of our results. Nevertheless, a larger analysis in the future has to confirm our explanatory results. Also, larger analyses would also allow for a stratification according to ECT indications.

## Conclusion

Including the serum concentration of psychiatric drugs into ECT treatment may offer a novel approach to optimize ECT treatment and advance *precision medicine*. We suggest considering serum concentrations of norquetiapine during ECT treatment to prevent reduced seizure durations. However, an adequate serum concentration threshold of norquetiapine below which seizure duration may compromise treatment response has not yet been established.

Regarding the psi as marker previously linked to clinical benefit, our findings suggested that patients with higher sum serum concentrations of ami may benefit more from ECT compared to patients with lower serum concentrations; however, treatment outcome was not considered in our study.

For clozapine our results showed that despite the known seizure threshold-lowering effects, it is likely that when patients were treated with serum concentrations below and within the TRR no additional risk for prolonged seizure duration is present during ECT treatment.

In summary, the interplay between serum concentrations of psychiatric drugs and seizure quality markers during ECT treatment remains complex and needs further investigations. With our study, we provide first evidence that serum concentrations of ami, and quet may affect seizure quality.

## Supplementary Information

Below is the link to the electronic supplementary material.

Supplementary Material 1

## Data Availability

The datasets analyzed during the current study are available from the corresponding author on reasonable request.

## Abbreviations

ami: Amitriptyline
ari: Aripiprazole
cari: Cariprazine
cloz: Clozapine
CI: Confidence interval
dox: Doxepin
ECT: Electroconvulsive therapy
FDR: False Discovery Rate
MPR: Metabolite-to-parent ratios
mirt: Mirtazapine
olanz: Olanzapine
psi: Postictal suppression index
quet: Quetiapine
risp: Risperidone
SD: Standard deviation
TDM: Therapeutic drug monitoring
ven: Venlafaxine
