# Supplementary material for: Association between serum concentrations of psychotropic drugs and seizure quality during ECT treatment
Source: BMC Psychiatry. 2026 Mar 17;26:290. doi: 10.1186/s12888-026-07981-7 (PMC13063754; doi:10.1186/s12888-026-07981-7)
Supplement: Supplementary file 1 — Supplementary Material 1 [file 12888_2026_7981_MOESM1_ESM.pdf]

Supplement to

Association between serum concentrations of psychotropic drugs and seizure quality during ECT treatment

Short running title: ECT and serum concentrations

Maike Scherf-Clavel, PhD[1#], Georg C. Ziegler, MD[1], Michael von Broen, MD[1], Sebastian Walther, MD[1]

[1] Department of Psychiatry, Psychosomatics and Psychotherapy, Center of Mental Health, University Hospital of Würzburg, 97080 Würzburg, Germany

[#]Corresponding author:

PD Dr. rer. nat. Maike Scherf-Clavel
Department of Psychiatry, Psychosomatics and Psychotherapy
University Hospital of Würzburg
Margarete-Höppel-Platz 1
97080 Würzburg, Germany
Tel.: +49/931/201 77546
Fax: +49/931/201 77262
E-Mail: Scherf_M@ukw.de

Supplemental Table 1 Full summary of all comorbidities in the sample. As patients are diagnosed with more than one diagnosis, number of psychiatric diagnoses exceed the number of patients/ECT sessions.

**Diagnoses**	**ICD-10 Code**	**N**
Recurrent depressive disorder, current episode severe without psychotic symptoms	F33.2	46
Bipolar affective disorder, current episode severe depression without psychotic symptoms	F31.4	13
Recurrent depressive disorder, current episode severe with psychotic symptoms	F33.3	11
Schizoaffective disorder, depressive type	F25.1	10
Severe depressive episode without psychotic symptoms	F32.2	7
Catatonic schizophrenia	F20.2	6
Bipolar affective disorder, current episode severe depression with psychotic symptoms	F31.5	6
Acute alcohol intoxication	F10.0	4
Schizoaffective disorder, mixed type	F25.2	4
Bipolar affective disorder, current episode mixed	F31.6	4
Compulsive acts	F42.1	4
Post-traumatic stress disorder (PTSD)	F43.1	4
Hypochondriacal disorder	F45.41	4
Attention-deficit hyperactivity disorder, predominantly inattentive type	F90.0	4
Alcohol dependence	F10.2	3
Harmful use of tobacco	F17.1	3
Tobacco dependence	F17.2	3
Generalized anxiety disorder	F41.1	3
Borderline personality disorder	F60.31	3
Paranoid schizophrenia	F20.0	2
Hebephrenic schizophrenia	F20.1	2
Bipolar affective disorder, manic episode with psychotic symptoms	F31.2	2
Panic disorder	F41.0	2
Mixed obsessional thoughts and acts	F42.2	2
Delirium not induced by dementia, due to alcohol or drugs	F05.0	1
Other delirium	F05.8	1
Organic mental disorder with psychiatric symptoms	F06.7	1
Alcohol withdrawal syndrome	F10.3	1
Harmful use of cannabis	F12.1	1
Harmful use of sedatives or hypnotics	F13.1	1
Dependence on sedatives or hypnotics	F13.2	1
Harmful use of multiple substances	F19.1	1
Manic episode with psychotic symptoms	F30.2	1
Bipolar affective disorder, hypomanic episode	F31.0	1
Bipolar affective disorder, manic episode without psychotic symptoms	F31.1	1
Other bipolar affective disorders	F31.8	1
Severe depressive episode with psychotic symptoms	F32.3	1
Social phobia	F40.1	1
Obsessive thoughts	F42.0	1

Dissociative stupor	F44.2	1
Somatization disorder	F45.1	1
Atypical eating disorder	F50.08	1
Nonorganic sleep disorder	F50.4	1
Mild intellectual disability, unspecified	F70.9	1
Chronic motor or vocal tic disorder	F95.1	1

Supplemental Table 2 Full summary of all drugs across the ECT sessions.

Drug	N
Quetiapine	173
Amitriptyline	155
Risperidone	119
Lorazepam	98
Lithium	84
Mirtazapine	81
L-Thyroxine	76
Venlafaxine	67
Ramipril	66
Bisprolol	52
Olanzapine	49
Atorvastatine	45
Clozapine	43
Pantoprazole	43
Acetylsalcylic acid	38
Aripiprazole	38
Doxepin	33
Cariprazine	31
Sertraline	31
Pirenzepine	28
Valproic acid	27
Amlodipine	25
Bupropion	25
Pregabaline	25
Valsartan	24
Metoprolol	23
Insulin	22
Metformine	21
Pipamperone	20
Duloxetine	18
Esomeprazole	18
Empagliflozin	17
Clomipramine	16
Chlorprothixen	15
Tamsulosine	15
Ezetimib	14
Propranolol	14
Trazodone	14
Ibuprofen	12
Temazepam	12
Torasemide	11
Benperidole	10
Novaminsulfone	10
Paracetamole	10
Spironolactone	10

Finasterid	9
Flupentixole	9
Haloperidole	8
Lamotrigine	8
Methylphenidate	8
Perazine	8
Irbesartane	7
Methionin	7
Nifedipine	7
Rivaroxaban	7
Simvastatine	7
Doxazosine	6
Allopurinole	5
Amphotericin B	5
Apixaban	5
Biperidenhydrochloride	5
Diazepam	5
Escitalopram	5
Ketokonazole	5
Prednisolone	5
Amiodarone	4
Distigminbromide	4
Paroxetine	4
Perphenazine	4
Aciclovir	3
Clopidogrel	3
Oxcarbazepine	3
Rosuvastatine	3
Silodosine	3
Tapentadole	3
Verapamil	3
Amisulpride	2
Betamethasonvalerate	2
Candesartane	2
Carvedilol	2
Lisdexamphetamine	2
Miconazole	2
Paliperidone	2
Promethazin	2
Sitagliptine	2
Sulpiride	2
Tiapride	2
Tolterodine	2
Ziprasidone	2
Zolpidem	2
Acetylcysteine	1
Agomelatine	1

Chlortalidone	1
Dimenhydrinate	1
Dulaglutide	1
Eplerenon	1
Etoricoxib	1
Fluoxetine	1
Hydrochlorothiazide	1
Metotrexat	1
Mirabegron	1
Prothipendyl	1

Supplemental Table 3 Number of patients and ECT sessions included in each model (seizure duration and psi).

	**seizure duration**		**psi**	
	patients (N=)	ECT sessions (N=)	patients (N=)	ECT sessions (N=)
Amitriptyline	33	126	32	97
Quetiapine	38	103	34	69
Risperidone	32	75	30	58
Venlafaxine	22	68	20	45
Mirtazapine	22	64	21	44
Clozapine	8	42	7	18
Olanzapine	15	41	13	32
Aripiprazole	18	40	13	29
Doxepin	10	30	9	19

Supplemental Table 3 Number of ECT sessions for each drug with available serum concentrations. Due to power reasons, only for groups with at least N=30, statistics were evaluated.

Drug	N
Amitriptyline	130
Quetiapine	106
Risperidone	77
Venlafaxine	68
Mirtazapine	66
Clozapine	42
Olanzapine	41
Aripiprazole	40
Doxepin	30
Cariprazine	30
Bupropion	29
Sertraline	27
Valproic acid	21
Clomipramine	18
Duloxetine	17
Trazodone	14
Flupentixol	9
Perazine	9
Lamotrigine	8
Benperidol	7
Pipamperon	7
Haloperdol	5
Escitalopram	4
Paroxetine	4
Perphenazine	4
Ziprasidone	2
Fluoxetine	1
Amisulpride	1

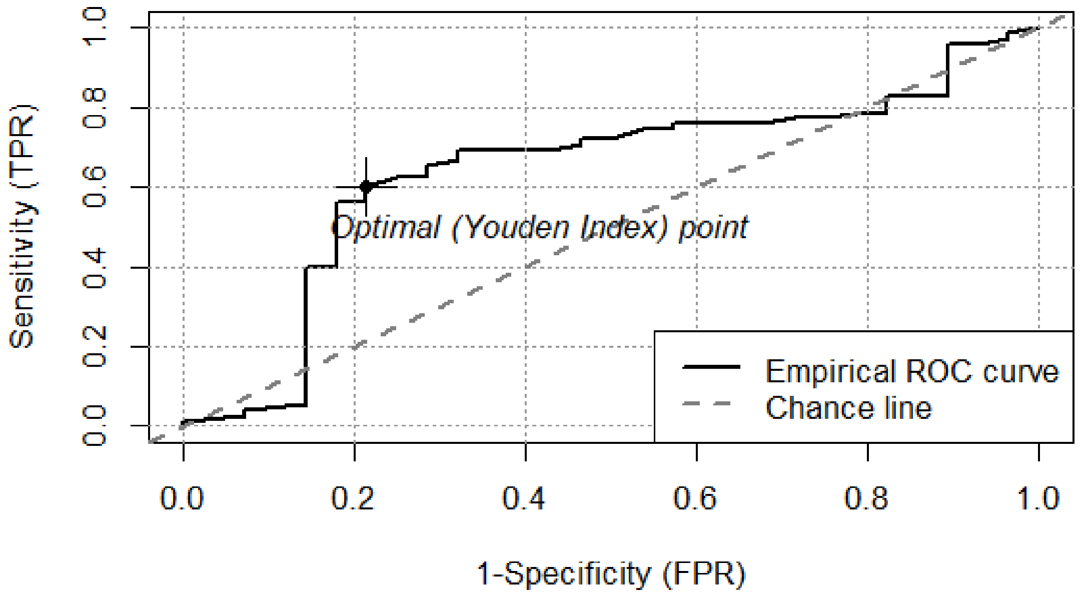

Supplemental Figure 1 ROC curve predicting seizure duration < 20 seconds using serum concentration of norquetiapine as predictor. Serum concentration was computed as 76 ng/mL (p=0.03; specificity 78.6%, sensitivity 60.0%). The area under the ROC curve was 0.6405 (95% CI 0.5157-0.7652). TPR, true positive rate; FPR, false positive rate
